# A Solitary Pedunculated Nevus Lipomatosus Superficialis

**DOI:** 10.7759/cureus.82040

**Published:** 2025-04-10

**Authors:** Julia S Murphy, John J Fowler, Morteza Khodaee

**Affiliations:** 1 Family Medicine, University of Colorado School of Medicine, Aurora, USA; 2 Pathology, University of Colorado School of Medicine, Aurora, USA; 3 Sports Medicine, University of Colorado School of Medicine, Aurora, USA

**Keywords:** acrochordon, excisional biopsy, lipofibroma, lipoma, pedunculated mass

## Abstract

Large pedunculated skin lesions are a rare presentation in primary care settings. In general, these lesions are asymptomatic other than occasional irritation and discomfort. Although differential diagnosis mainly includes benign lesions, an excisional biopsy should be performed, particularly in symptomatic cases. We present the case of a woman in her 40s with an irritating large and pedunculated skin lesion on her back. Due to the symptomatic nature of the lesion, an excisional biopsy was performed, which confirmed the diagnosis of a nevus lipomatosus superficialis.

## Introduction

Pedunculated skin lesions are attached to the body by an elongated stalk known as a peduncle. Although pedunculated skin lesions are common, the majority of them are small in size. Large pedunculated skin lesions are rare [1,2]. A well-vascularized stem is necessary for a pedunculated skin lesion to grow large [2]. These lesions typically grow slowly over several years [1-4]. While the majority of large pedunculated skin lesions are benign, there are rare instances where premalignant or malignant lesions may be present [1-5]. Benign conditions that can appear as large pedunculated cutaneous lesions include acrochordon, fibroepithelial polyps, lipoma, neurofibroma, epidermal nevus, fibrous histiocytoma, nevus lipomatosus superficialis (NLS) (lipofibroma), pyogenic granuloma, dermatofibroma, cutaneous mixed tumor, cylindroma, acquired fibrokeratoma, schwannoma, cutaneous myxoma, and cutaneous angiomyxoma [1,2]. Although extremely rare, malignant lesions presenting as pedunculated cutaneous growths may include basal cell carcinoma, melanoma, Merkel cell carcinoma, cutaneous sarcoma, and pilomatrix carcinoma [2]. While the differential diagnosis primarily involves benign lesions, an excisional biopsy should be performed, especially in cases with an unusual pattern or symptoms [1-4,6,7]. The majority of patients seek medical attention for cosmetic reasons. In this case, we present the case of a woman with a large pedunculated skin lesion on her back that had been present for several years.

## Case presentation

**Figure 1:**
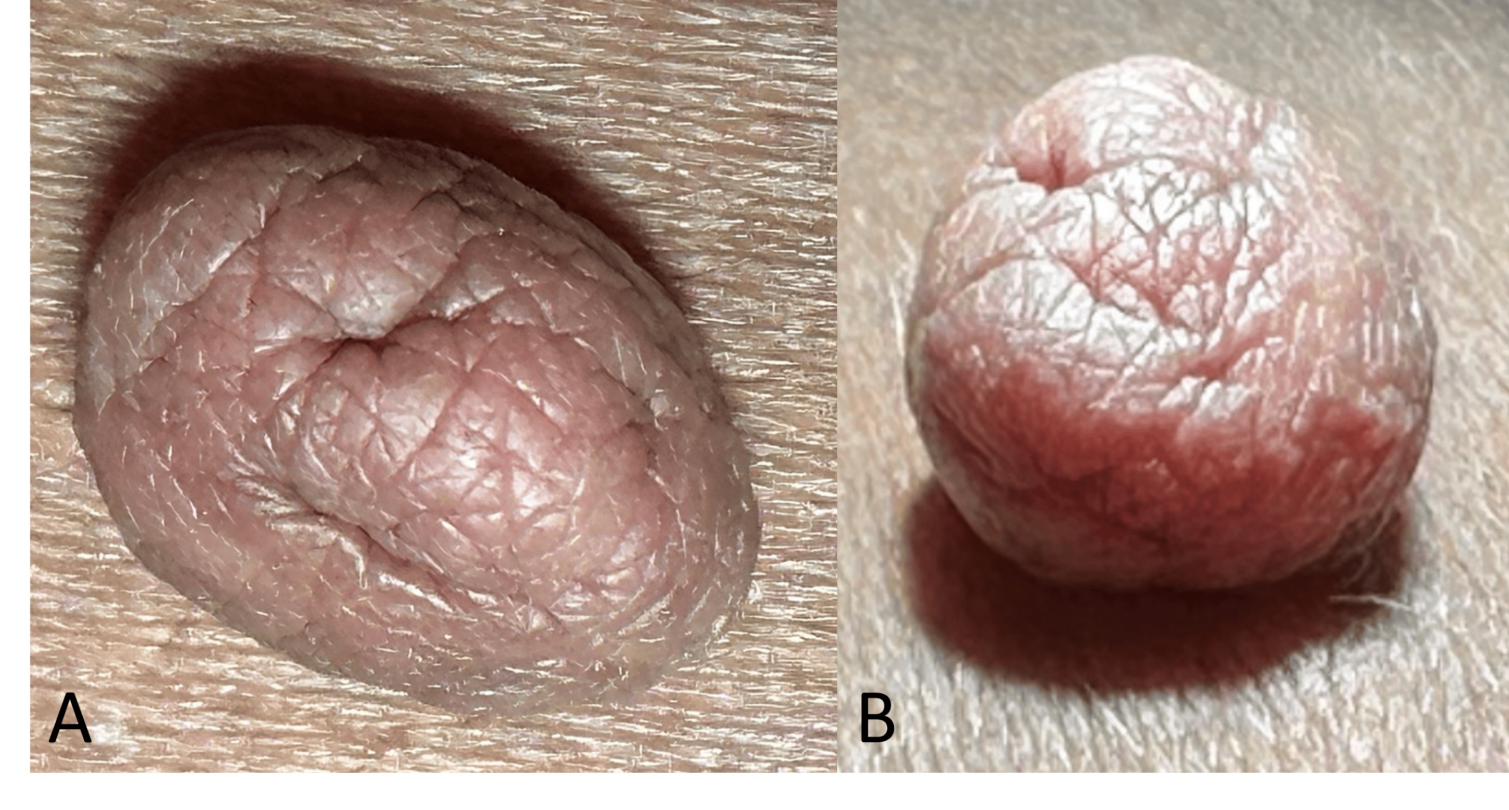
A flesh-colored, pedunculated skin lesion with cerebriform surface (A and B) on the left flank that was 1.4 × 1.1 × 0.8 cm in size, soft, mobile, and non-tender.

A woman in her 40s presented with an enlarging skin lesion on her left lower back. The lesion had been growing over the last two to three years and was cosmetically bothersome to her. The lesion does not cause any pain, but it may occasionally get caught, particularly when changing clothes. On physical examination, there was a 1.4 x 1.1 x 0.8 cm, non-tender, pedunculated, flesh-colored cerebriform lesion on the left lower back (Figures 1A, 1B).

**Figure 2:**
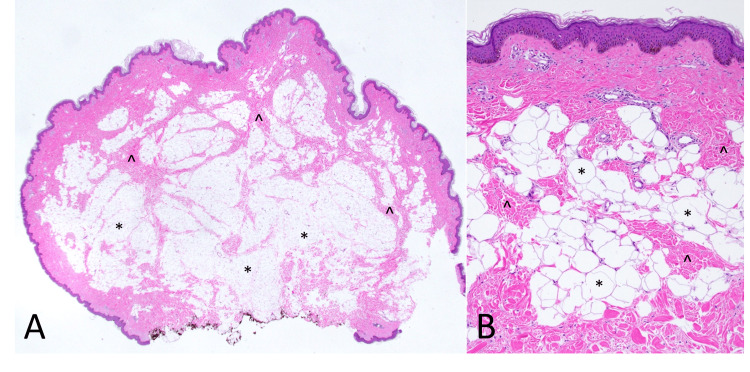
Histologic sections (A) demonstrate a polypoid lesion formed by lobules of adipocytes (*) within the reticular dermis. Bands of dermal collagen course (^) throughout the lesion (H&E, 12.5× magnification). On higher magnification (B), bands of dermal collagen (^) are intercalated among collections of mature adipocytes (*), which are in close proximity to the epidermis (H&E, 100× magnification). H&E: hematoxylin and eosin

After discussing options with the patient, she elected to have the lesion removed. An excisional biopsy was performed, and the sample was sent for pathological evaluation. Histopathology confirmed the diagnosis of a pedunculated NLS (Figures 2A, 2B).

## Discussion

NLS, also known as pedunculated lipofibroma, is a rare benign hamartoma characterized by the presence of adipose tissue in the dermis [3-8]. The etiology of NLS is unclear [6]. NLS affects both men and women equally, with no preference for either sex [4,6,8-11]. There have also been some case series suggesting an association with obesity and/or diabetes, but further research is necessary [5,6]. NLS has been categorized into the classic multiple form and the solitary variant [3-12]. In rare cases, NLS can ulcerate, especially in cases with irritation [4,6,8]. The classical type generally presents near birth and consists of multiple soft papulonodules and sometimes plaques [3-12]. The solitary type is more common in areas of pressure similar to acrochordons [4-8]. The solitary type is typically larger in size and appears as a single pedunculated skin lesion [4,5,7,12]. Aside from differences in their sizes, anatomical locations, and the number of lesions, both types exhibit similar clinical and histopathological features [5,6].

However, some have proposed classifying these two types as distinct entities [5,6]. Clinically, these lesions may have a smooth surface or multilobular (cerebriform) surface with prominent folds [5,6,8]. Histopathologically, NLS is characterized by mature adipose tissue within the dermis [3,5,6,8]. The recommended treatment is surgical excision, and the lesions typically do not recur [3-6,8]. Although these lesions appear benign, it is strongly recommended to send the excised samples for pathological evaluation [1,2,4,5,8,10,11].

## Conclusions

Large pedunculated skin lesions are rare findings in primary care settings. These lesions are typically asymptomatic but may occasionally cause irritation or discomfort. Although the differential diagnosis often includes benign lesions, malignancy should also be considered. An excisional biopsy is recommended, especially for lesions with unusual features, those causing irritation, or those removed for cosmetic reasons.
